# Correction: Kallikrein Gene-Modified EPCs Induce Angiogenesis in Rats with Ischemic Hindlimb and Correlate with Integrin αvβ3 Expression

**DOI:** 10.1371/annotation/67f865b9-41af-4fd7-a9af-e06078cb4f75

**Published:** 2013-10-01

**Authors:** Shen Shen Fu, Fu Ji Li, Yuan Yuan Wang, An Bei You, Yi Liang Qie, Xiao Meng, Jian Rui Li, Bao Chuan Li, Yun Zhang, Qing Da Li

All authors in the article are also affiliated with: The Key Laboratory of Cardiovascular Remodeling and Function Research, Chinese Ministry of Education and Chinese Ministry of Health, Shandong University Qilu Hospital, Jinan, Shandong Province, China 

